# Physical activity and adolescents' parasocial relationships with virtual idols: evidence from loneliness, gender differences, and network analysis

**DOI:** 10.3389/fpsyg.2026.1854829

**Published:** 2026-06-30

**Authors:** Longjiang Chen, Ziyang Lin, Zeyan Wang, Jiale Wang, Yian Xiang

**Affiliations:** 1 Institute of Physical Education, Yunnan Normal University, Kunming, China; 2 College of Sport and Art, Shenzhen Technology University, Shenzhen, China; 3 School of Physical Education, Chengdu University of Technology, Chengdu, China; 4 College of Physical Education, Xinjiang Normal University, Ürümqi, Xinjiang, China; 5 Department of Rehabilitation Medicine, Lishui Municipal Central Hospital, The Fifth Affiliated Hospital of Wenzhou Medical University, Lishui, China

**Keywords:** adolescents, loneliness, network analysis, parasocial relationship, physical activity, virtual idols

## Abstract

**Introduction:**

This study aimed to examine whether higher levels of physical activity were associated with adolescents' parasocial relationships with virtual idols through lower levels of loneliness, and to determine whether gender moderated these associations. The study also explored the item-level connections between loneliness and parasocial relationships with virtual idols.

**Methods:**

A cross-sectional survey was conducted among 2,798 adolescents from Sichuan, Hunan, Yunnan, and Chongqing provinces in China. Physical activity, loneliness, and parasocial relationships with virtual idols were measured using validated questionnaires. The SPSS PROCESS macro was used to test the moderated mediation model, and network analysis was conducted to identify key bridging nodes between loneliness and parasocial relationships.

**Results:**

Physical activity was significantly negatively associated with parasocial relationships with virtual idols, and loneliness showed a significant partial statistical mediation in this association. Gender significantly moderated both the association between physical activity and loneliness and the association between loneliness and parasocial relationships, with these associations being more pronounced among female adolescents. Network analysis further identified bridging connections between real-life relational deficits, such as feeling neglected and lacking companionship, and emotional involvement with virtual idols, such as caring about the life of a virtual idol.

**Discussion:**

The findings suggest that physical activity may be associated with weaker parasocial relationships with virtual idols not only through time occupation or attention diversion, but also through lower loneliness and stronger real-life social connection. Interventions aimed at reducing excessive emotional dependence on virtual idols should therefore focus on promoting physical engagement, real-life interaction, and adolescents' sense of belonging.

## 1 Introduction

As traditional social connections weaken and the process of individualization accelerates, issues related to loneliness and social alienation have gained increasing attention in modern society (Holt-Lunstad, 2024; Jeste et al., 2020). In this context, virtual idols have gradually become a significant object of media interaction and emotional projection for young people (Hu and Xiao, 2023; Liu, 2025; Stein et al., 2024). Virtual idols are entertainment figures created and presented in virtual spaces using digital media technologies. Their images, behaviors, and performances are typically generated through technologies such as digital modeling, motion capture, holographic projection, and artificial intelligence (Yu and Geng, 2020). Unlike real idols, virtual idols do not depend on specific biological individuals but are sustained through technology that enables continuous content output and image shaping. Fans, through prolonged contact and interaction with virtual idols, gradually form a one-sided yet emotionally invested psychological connection, a phenomenon commonly defined as the parasocial relationship between virtual idols and fans (PSR) (Hoffner and Bond, 2022). Existing research suggests that PSR can provide individuals with a sense of companionship, identification, and emotional comfort (Breves and Van Berlo, 2025; Liu, 2025). However, when individuals primarily rely on virtual idols to compensate for emotional deficiencies and belonging needs in real life, an overly strong parasocial relationship may pose potential risks. For example, when individuals become excessively involved in parasocial relationships with virtual idols, they may redirect psychological resources that should be invested in real-life learning, social interactions, and emotional regulation toward virtual entities, thereby reducing interpersonal contact and emotional exchange in the real world (Cheng and Zhang, 2025; Diaz et al., 2026). In such cases, individuals are more likely to experience temporary feelings of companionship and emotional comfort through attention, following, and imaginary interactions, but their real-life belonging needs may not be genuinely fulfilled. If individuals continue to substitute virtual relationships for real-life connections over time, their emotional support may remain at an imaginary or mediated level, potentially exacerbating loneliness and impeding real social connections and interpersonal adaptation (Liu, 2024; Seidler et al., 2022). This is particularly concerning for groups with weaker psychological resources or insufficient real-life connections, where parasocial relationships with virtual idols may develop into compensatory emotional dependencies, further limiting their opportunities to gain stable support and a sense of belonging from real social relationships (He and Sun, 2022). Therefore, investigating the formation mechanisms and influencing factors of parasocial relationships with virtual idols holds significant theoretical and practical value.

Existing research on parasocial relationships with virtual idols mainly focuses on factors such as the media characteristics of virtual idols, their attractiveness, perceived interactivity, and fan culture, with relatively limited attention given to social participation and social connection factors in individuals' real lives. However, the formation of parasocial relationships with virtual idols not only depends on the media objects themselves but is also influenced by individuals' real-life social situations, such as their loneliness, the degree to which their belonging needs are met, and their social connection levels. Thus, an explanation that solely focuses on the media aspect is insufficient (Liu, 2025; Stein et al., 2024; Yu et al., 2025). In contrast to relying primarily on online media relationships for emotional satisfaction, physical activity (PA) may provide adolescents with opportunities for bodily involvement, real-life participation, and contextual engagement. From the perspective of Self-Determination Theory, the psychological significance of physical activity depends not only on participation itself, but also on whether the activity context supports adolescents' basic psychological needs for relatedness, competence, and autonomy (Tsai et al., 2021). Activities involving peers, coaches, teams, shared rules, or collective goals may strengthen relatedness by providing real-life interaction, shared participation, and a sense of being involved with others. More solitary forms of physical activity may still support competence and autonomy through self-chosen goals, bodily mastery, and perceived control, although their connection with loneliness through social relatedness may be less direct. Systematic reviews and original studies have indicated that physical activity interventions may be associated with lower loneliness under certain conditions, partly through improved social experiences and positive social interactions (Ahn et al., 2024; Park et al., 2025; Wang et al., 2025; Shvedko et al., 2018). Therefore, adolescents who engage in more need-supportive physical activity may be more likely to experience lower loneliness.

Loneliness (LN) refers to an individual's subjective perception that the quantity or quality of real-life social relationships is insufficient (Jeste et al., 2020). From the perspective of belonging needs and compensatory media use, individuals who are unable to obtain sufficient intimate relationships, emotional responses, and belonging experiences in real life are more likely to turn to media relationships that are easier to access and involve lower interpersonal risk to satisfy their social and emotional needs (Akinci and Durmuş, 2025). Virtual idols may be particularly suitable for this compensatory function because their images are stable, their content is continuously available, and emotional projection can occur with relatively low social cost. In this sense, adolescents with lower levels of loneliness may have less need to compensate for unmet relational needs through emotional involvement with virtual idols, whereas adolescents with higher levels of loneliness may be more likely to form stronger parasocial relationships with virtual idols. Existing parasocial research also suggests that unmet interpersonal needs, including loneliness, are important psychological drivers of parasocial relationships. Studies focused on virtual idol fans have found that loneliness is positively associated with parasocial relationships between fans and virtual idols (Greenwood and Aldoukhov, 2023; Kim et al., 2023). Moreover, numerous studies have demonstrated a stable negative relationship between physical activity and loneliness, and intervention studies have also indicated that physical activity can help alleviate loneliness (Ahn et al., 2024; Gen et al., 2025). Taken together, individuals with higher levels of physical activity may experience lower levels of loneliness and may therefore be less reliant on the compensatory emotional connections provided by virtual idols. In contrast, individuals with lower levels of physical activity may experience higher levels of loneliness, making them more likely to form stronger parasocial relationships with virtual idols.

In addition to the mediating mechanism, gender may serve as an important boundary condition because the proposed pathway is relational in nature. Gender socialization and relationship orientation perspectives suggest that male and female adolescents may differ in emotional expression, relationship cue processing, and sensitivity to interpersonal support (Wang et al., 2008). Previous studies have also shown that parasocial processes can exhibit gender differences, including differences in preferences for media figures, reasons for sustained attention, and modes of emotional involvement (Gleason et al., 2017). Female adolescents may be more responsive to relational support and emotional feedback in physical activity, which could make the association between physical activity and loneliness stronger among females. This interpretation is also consistent with evidence suggesting that the relationship between physical activity and loneliness may vary across gender groups (Nakajima et al., 2025; Nie et al., 2025). At the same time, when real-life relational needs are not sufficiently met, loneliness may be more likely to translate into emotional involvement with virtual idols among females because virtual idols can provide stable, low-risk, and symbolically responsive companionship. Therefore, gender was expected to moderate the association between physical activity and loneliness and the association between loneliness and parasocial relationships with virtual idols.

Traditional variable-centered analysis, while capable of examining the relationship between loneliness and parasocial relationships with virtual idols, still faces limitations in addressing a more nuanced question: what specific psychological components drive these effects (Hofmann et al., 2020). For complex psychological phenomena like parasocial relationships with virtual idols, relying solely on overall scores may compress inherently heterogeneous components into a single index, thus diminishing the ability to identify micro-level mechanisms. Item network analysis allows for a further refinement of the relationships between variables, breaking them down into conditional associations between items and identifying key and bridging nodes within the network. This method can reveal more critical connections between deficiencies in real-life relationships and emotional investment in virtual idols. Therefore, in addition to variable-level mediation and moderated mediation analyses, the introduction of item network analysis helps uncover the internal relational structure between loneliness and parasocial relationships with virtual idols at a finer level, providing supplementary evidence for the proposed mechanisms.

**Figure 1:**
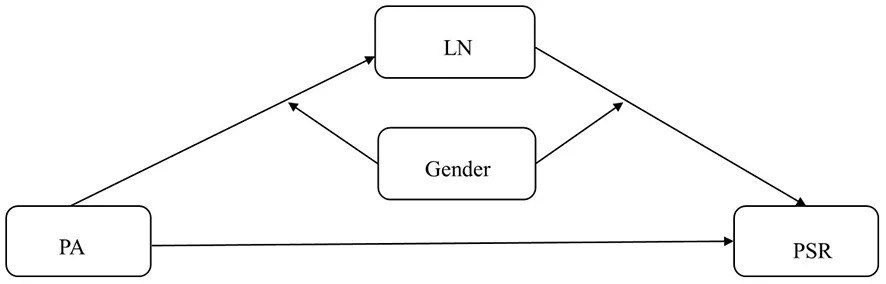
Schematic diagram of the hypothetical model.

In summary, while existing research has identified the connection between parasocial relationships with virtual idols and loneliness and has accumulated substantial evidence linking physical activity to lower loneliness, several gaps remain. First, research on parasocial relationships with virtual idols has predominantly focused on media attributes and idol characteristics, with limited attention given to real-life lifestyle variables such as physical activity. Second, existing studies rarely provide a systematic explanation of how real-life protective factors influence parasocial relationships via psychological mechanisms, and there is insufficient investigation of whether gender differences exist in these processes. Finally, much of the research has remained at the overall score level, examining variable relationships, with limited focus on the internal connections at the item level between loneliness and parasocial relationships with virtual idols, as well as the identification of key bridging components. Based on these gaps, this study aims to examine the association between physical activity and parasocial relationships with virtual idols from the perspective of real-life lifestyle, while further testing the statistical mediating role of loneliness and the moderating role of gender. Additionally, item network analysis will be employed to identify the critical connection components between loneliness and parasocial relationships with virtual idols. This research not only contributes to expanding the explanatory framework of parasocial relationships with virtual idols but also provides empirical evidence for repairing real-life relationships and promoting mental health for adolescents who experience higher levels of loneliness and deeper emotional involvement with virtual idols. Based on this, the following hypotheses and model are proposed (see Figure 1):

**H1**: Physical activity are negatively associated with parasocial relationships with virtual idols.

**H2**: Loneliness statistically mediates the association between physical activity and parasocial relationships with virtual idols.

**H3**: Gender moderates the mediation pathway linking physical activity to parasocial relationships with virtual idols through loneliness.

## 2 Research instruments

### 2.1 Participants

This cross-sectional study focused on adolescent students as the research subjects. Data collection was conducted from September 2025 to November 2025 in four regions of China: Sichuan, Hunan, Yunnan, and Chongqing. Adolescents who signed informed consent forms and obtained parental or guardian permission were eligible to complete the questionnaire. To ensure data quality, a strict cleaning process was implemented on the respondents' answers. Following the recommendations of DeSimone et al. (2015) and Meade and Craig (2012) for identifying low-quality responses, a three-stage cleaning procedure was applied. First, responses completed in less than 120 s were excluded, as this time frame typically indicates a lack of attention. Second, answers identified as inconsistent based on logical checks between items were deleted. Finally, questionnaires with consecutive identical responses were discarded. A total of 2,956 questionnaires were collected. After data cleaning, invalid questionnaires and explanatory records were removed, resulting in 2,798 valid samples, yielding a response rate of 94.7%. Among the valid participants, 1,472 were male (52.6%) and 1,326 were female (47.4%). The majority of the sample consisted of middle school students, with 812 in the 1^st^ year (Grade 7), 714 in the 2^nd^ year (Grade 8), and 866 in the third year (Grade 9), accounting for 85.5% of the total. High school students included 148 in the 1^st^ year (Grade 10), 154 in the 2^nd^ year (Grade 11), and 104 in the 3^rd^ year (Grade 12), comprising 14.5% of the sample. The mean grade level was 2.44 with a standard deviation of 1.31, indicating that the sample was predominantly composed of middle school students. In terms of background variables, 984 students were from urban areas (35.2%) and 1,814 from rural areas (64.8%). The mean for the urban-rural variable was 1.65, with a standard deviation of 0.48. Regarding family structure, 354 were only children (12.7%) and 2,444 were non-only children (87.3%), with a mean of 1.87 and a standard deviation of 0.33 for the only-child variable. Additionally, 660 were left-behind children (23.6%) and 2,138 were not (76.4%), with a mean of 1.76 and a standard deviation of 0.42 for the left-behind variable.

### 2.2 Measurement tools

Physical activity participation was assessed using the Physical Activity Level Scale developed by Liang (1994), which measures the frequency, intensity, and duration of adolescents' participation in physical activity over the past month. The scale consists of three items and employs a 1–5 rating system. The total score for physical activity is calculated as: Total Physical Activity Score = Frequency × Intensity × (Time - 1). The total score ranges from 0 to 100, with scores ≤ 19 indicating low physical activity, 20–42 indicating moderate physical activity, and ≥43 indicating high physical activity. Higher scores indicate greater levels of physical activity. In this study, the Cronbach's α coefficient for the scale was 0.782, with a KMO value of 0.762. Loneliness was measured using the UCLA loneliness Scale (revised version) developed by Wongpakaran et al. (2020) through Rasch analysis, designed to assess the level of loneliness in individuals. This scale is unidimensional, consisting of 6 items rated on a 4-point scale, ranging from 1 (never) to 4 (always). The total score ranges from 6 to 24, with higher scores indicating greater levels of loneliness. Example items include: “Do you feel a lack of companionship?” and “Do you feel left out?” In this study, the Cronbach's α coefficient for the scale was 0.851, with a KMO value of 0.869.

Th**e** parasocial relationship between virtual idols and fans was assessed based on Liu's (2025) scale for measuring the degree of parasocial relationships between individuals and virtual idols. This scale includes three dimensions—cognitive, emotional, and behavioral—comprising 15 items. Example items include: “I deeply care about her work and all aspects of her life” and “I sometimes imagine her as my friend or intimate partner. “Items are rated on a 5-point Likert scale, where 1 indicates 'strongly disagree,' and 5 indicates 'strongly agree.” Higher scores reflect a stronger attachment to the virtual idol. In this study, the Cronbach's α coefficient for the scale was 0.708, with a KMO value of 0.957.

### 2.3 Data processing

In this study, SPSS 27.0 **s**oftware was used to perform descriptive statistics, Pearson correlation analysis, and reliability and validity tests for the main variables. Based on these analyses, the PROCESS 4.2 macro developed by Hayes (2013) was employed to further examine the relationships between the variables. Specifically, Model 4 was used to examine the statistical mediation model, which estimates the indirect association between the independent variable and the dependent variable through the mediator. Model 58 was used to examine the conditional process model, assessing whether the associations from the independent variable to the mediator and from the mediator to the dependent variable differed by the moderator. All effects were estimated using 5,000 bootstrap samples to compute 95% confidence intervals. If the confidence interval did not include 0, the effect was considered significant. To further explore the structural associations between loneliness and the parasocial relationship between virtual idols and fans at the item level, network analysis was conducted using the bootnet, qgraph, networktools, and NetworkComparisonTes**t** packages in R 4.5.2. Node strength, bridge strength, and bridge expected influence were calculated, and the accuracy and stability of the network were assessed through bootstrap testing. Additionally, exploratory network comparisons by gender were conducted to investigate potential differences between male and female groups.

## 3 Results

### 3.1 Common method bias

Common method bias was tested using Harman's single-factor test (Podsakoff and Organ, 1986). The results showed that the four factors extracted without rotation, which had eigenvalues greater than one, indicated that the first factor explained 38.71% of the variance (less than 40%). This suggests that common method bias did not significantly affect the results of this study.

### 3.2 Descriptive statistics and correlation analysis

Table 1 reports the descriptive statistics and correlation analysis results for the main variables. The results indicate that physical activity were significantly negatively correlated with loneliness (*r* = −0.316, *p* < 0.01) and with the parasocial relationship between virtual idols and fans (*r* = −0.365, *p* < 0.01). Loneliness was significantly positively correlated with the parasocial relationship between virtual idols and fans (*r* = 0.498, *p* < 0.01). Additionally, gender was significantly negatively correlated with physical activity (*r* = −0.310, *p* < 0.01), significantly positively correlated with loneliness (*r* = 0.169, *p* < 0.01), and significantly positively correlated with the parasocial relationship between virtual idols and fans (*r* = 0.134, *p* < 0.01). These results indicate significant correlations between the main variables, providing a statistical basis for subsequent mediation analysis.

**Table 1:** Descriptive statistics and correlation analysis (*n* = 2,798).

Variable	M	SD	1	2	3	4
1 PA	30.76	21.075	1			
2 LN	11.98	4.053	−0.316^**^	1		
3 PSR	43.28	8.249	−0.365^**^	0.498^**^	1	
4 gender	1.47	0.499	−0.310^**^	0.169^**^	0.134^**^	1

^**^ denotes *p* < 0.01. PA, physical activity; LN, loneliness; PSR, parasocial relationship between virtual idols and fans.

### 3.3 Statistical mediation analysis

**Figure 2:**
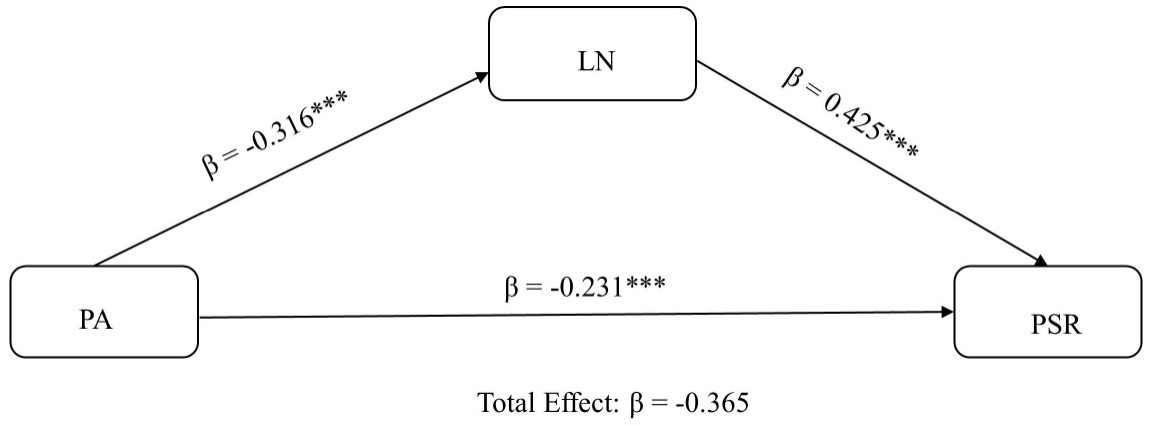
Statistical mediation model linking physical activity, loneliness, and parasocial relationships with virtual idols. *** denotes *p* < 0.001.

Before analysis, all variables were standardized. As shown in Table 2 and Figure 2, physical activity were significantly negatively associated with loneliness [β = −0.316, SE = 0.018, *t* = −17.631, *p* < 0.001, 95% CI (−0.352, −0.281)], suggesting that higher levels of physical activity were related to lower levels of loneliness. Further analysis showed that physical activity were significantly negatively associated with the parasocial relationship between virtual idols and fans [β = −0.231, SE = 0.017, *t* = −13.779, *p* < 0.001, 95% CI (−0.263, −0.198)], while loneliness was significantly positively associated with the parasocial relationship between virtual idols and fans [β = 0.425, SE = 0.017, *t* = 25.384, *p* < 0.001, 95% CI (0.392, 0.458)]. Additionally, the total association between physical activity and the parasocial relationship between virtual idols and fans was significant [β = −0.365, SE = 0.018, *t* = −20.727, *p* < 0.001, 95% CI (−0.400, −0.330)]. Bootstrap testing showed that the indirect association between physical activity and the parasocial relationship between virtual idols and fans through loneliness was significant [β = −0.134, BootSE = 0.009, 95% CI (−0.153, −0.117)]. The confidence interval did not contain 0, indicating that loneliness showed a significant partial statistical mediation in the association between physical activity and the parasocial relationship between virtual idols and fans.

**Table 2:** Statistical mediation analysis of physical activity, loneliness, and parasocial relationships with virtual idols.

Path/effect	β	SE	*t*	*p*	LLCI	ULCI
PA → LN	−0.316	0.018	−17.631	< 0.001	−0.352	−0.281
PA → PSR	−0.231	0.017	−13.779	< 0.001	−0.263	−0.198
LN → PSR	0.425	0.017	25.384	< 0.001	0.392	0.458
PA → PSR (total effect)	−0.365	0.018	−20.727	< 0.001	−0.4	−0.33
PA → LN → PSR (indirect effects)	−0.134	0.01	—	—	−0.153	−0.117

### 3.4 Moderated mediation effect

**Figure 3:**
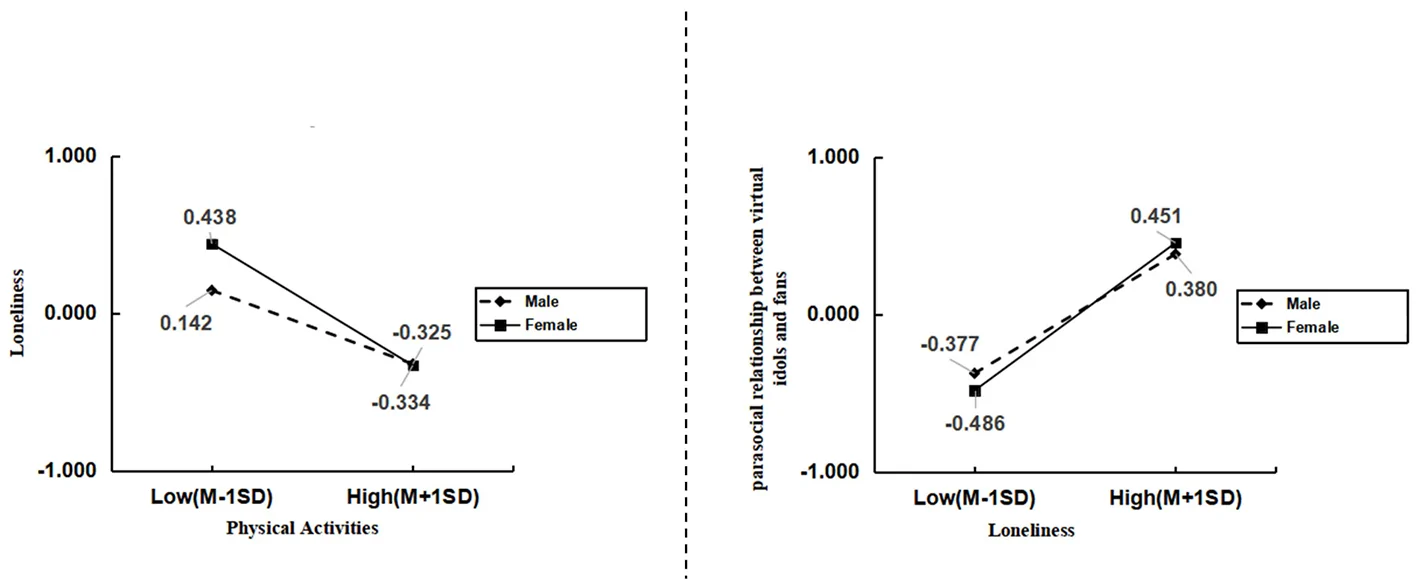
Simple slope plot of gender moderation.

To examine whether gender moderated the statistical association linking physical activity, loneliness, and parasocial relationships with virtual idols, physical activity was entered as the independent variable, loneliness as the mediator, parasocial relationships with virtual idols as the dependent variable, and gender as the moderator. This study used physical activity as the independent variable, loneliness as the mediator, and the parasocial relationship between virtual idols and fans as the dependent variable, with gender as the moderator. The results are shown in Table 3 and Figure 3. First, in the regression equation with loneliness as the dependent variable, gender was significantly positively associated with loneliness (β = 0.143, SE = 0.038, *t* = 3.797, *p* < 0.001). Physical activity were significantly negatively associated with loneliness (β = −0.306, SE = 0.019, *t* = −16.026, *p* < 0.001), indicating that higher levels of physical activity are associated with lower levels of loneliness. Furthermore, the interaction term between physical activity and gender was significantly associated with loneliness (β = −0.153, SE = 0.039, *t* = −3.958, *p* < 0.001), suggesting that gender moderated the association between physical activity and loneliness. The explanatory power of this regression model was 0.111, and the overall model was significant (*F* = 115.78, *p* < 0.001).

**Table 3:** Moderated mediation analysis results.

Variable	LN				PSR			
	β	SE	*t*	*p*	β	SE	*t*	*P*
Gender	0.143	0.038	3.797	< 0.001	−0.019	0.034	−0.579	0.563
PA	−0.306	0.019	−16.026	< 0.001	−0.234	0.017	−13.445	< 0.001
LN					0.421	0.017	25.002	< 0.001
PA × gender	−0.153	0.039	−3.958	< 0.001				
LN × gender					0.090	0.032	2.786	0.005
*R^2^*	0.111				0.298			
*F*	115.78			< 0.001	295.861			< 0.001

Second, in the regression equation with parasocial relationships with virtual idols as the dependent variable, gender was not significantly associated with parasocial relationships after the other variables were included in the model (β = −0.019, SE = 0.034, *t* = −0.579, *p* = 0.563). However, physical activity were significantly negatively associated with the parasocial relationship (β = −0.234, SE = 0.017, *t* = −13.445, *p* < 0.001), and loneliness was significantly positively associated with the parasocial relationship (β = 0.421, SE = 0.017, *t* = 25.002, *p* < 0.001). This suggests that higher levels of physical activity are associated with weaker parasocial relationships with virtual idols, while higher levels of loneliness are associated with stronger parasocial relationships. Additionally, the interaction term between loneliness and gender was significantly associated with the parasocial relationship (β = 0.090, SE = 0.032, *t* = 2.786, *p* = 0.005), indicating that gender moderated the association between loneliness and the parasocial relationship. The explanatory power of this regression model was 0.298, and the overall model was significant (*F* = 295.861, *p* < 0.001).

To further explain the interaction effect, a simple slope analysis was conducted, with the results shown in Figure 3. Regarding the association between physical activity and loneliness, as the level of physical activity increased, both males and females exhibited lower loneliness levels, with females showing a more pronounced decrease. This suggests that the negative association between physical activity and loneliness was stronger among females. Regarding the association between loneliness and parasocial relationships, as loneliness levels increased, both males and females showed stronger parasocial relationships, with females experiencing a greater increase. This indicates that the positive association between loneliness and parasocial relationships was stronger among females.

### 3.5 Network analysis results

**Figure 4:**
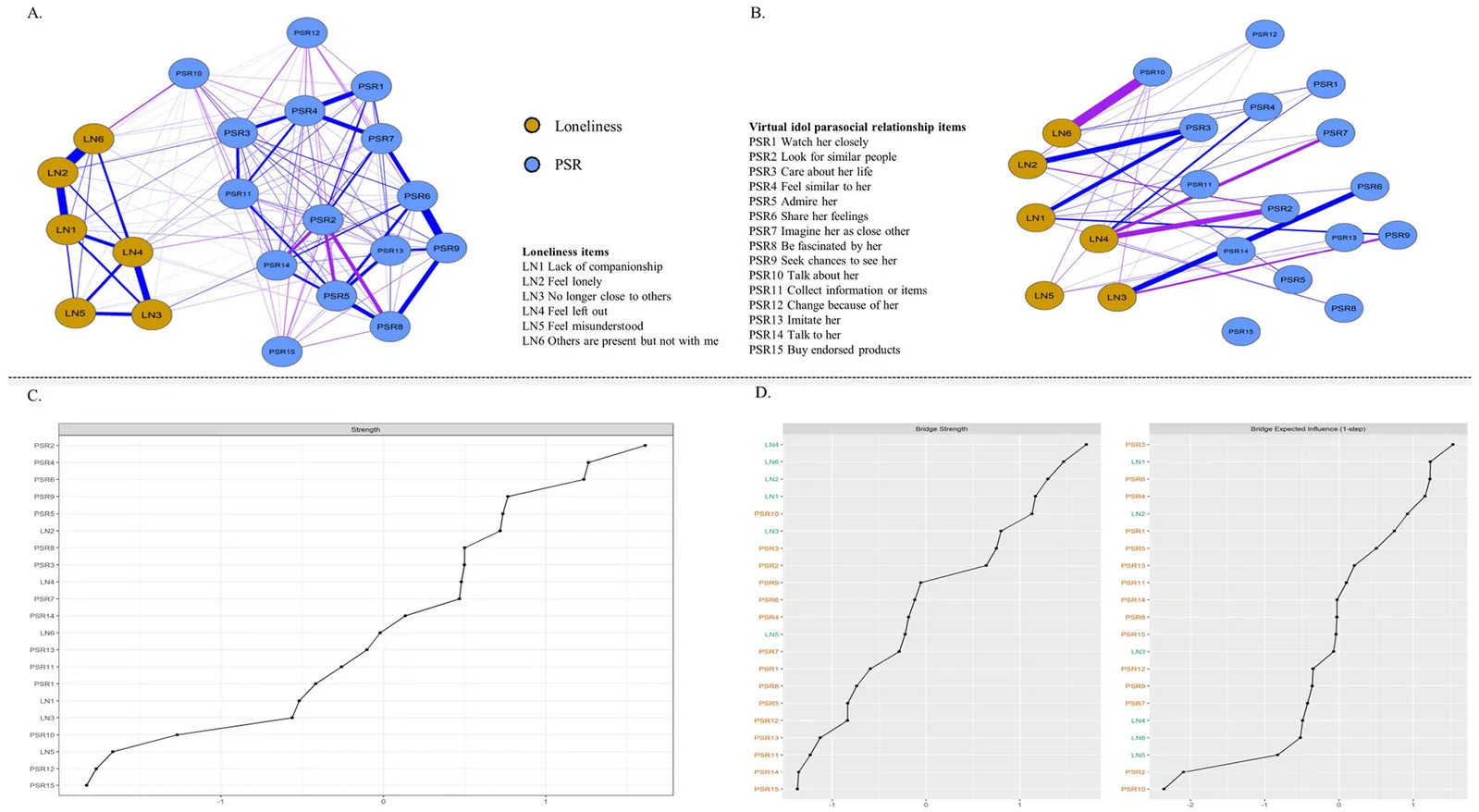
Item network structure, bridging characteristics, and centrality results of loneliness and the parasocial relationship between virtual idols and fans. **(A)** Overall network. **(B)** Bridge network. **(C)** Strength centrality. **(D)** Bridge indicators.

At the item level, a network consisting of 21 nodes was constructed based on the 2,798 valid samples after data cleaning. The network includes 6 nodes representing loneliness and 15 nodes representing the parasocial relationship between virtual idols and fans. After network estimation, 145 non-zero edges were retained, with 87 positive edges and 58 negative edges. The overall network is shown in Figure 4A. It is evident that the network does not exhibit a homogeneous, diffuse connection pattern, but rather forms two relatively distinct local modules centered around loneliness and the parasocial relationship between virtual idols and fans. In other words, the two types of items maintain strong aggregation within their respective communities but are not isolated from one another. Instead, they form substantial coupling through several cross-community connections. This result indicates that the relationship between loneliness and the parasocial relationship between virtual idols and fans is not limited to the total score level of variables but also reveals discernible structural connections at the more granular item level.

From the centrality results, as shown in Figure 4C, nodes such as “seeking people similar to her,” “feeling similar to her,” “sharing her feelings,” “looking for opportunities to meet her,” and “admiring her” are located in the relative center of the parasocial relationship between virtual idols and the fans network. At the same time, the node “feeling lonely” within loneliness also shows high node strength. This suggests that identity, emotional involvement, and proximity-related components in the parasocial relationship have a stronger organizational role within the network, while the core emotional components of loneliness form crucial support points that interlink with these components. Further examination of the bridging indicators reveals that the cross-community connections, shown in Figures 4B, 4D, are not evenly distributed across all nodes but are concentrated in a few entries with bridging functions. Specifically, nodes such as “feeling neglected,” “others are around me but not truly with me,” and “lack of companionship” in loneliness, as well as “talking about her” and “caring about her life” in the parasocial relationship, demonstrate more prominent bridging roles. It is not the abstract concept of loneliness itself, but rather the experiences of lack of companionship, feelings of neglect, and the absence of relational presence that bridge the gap between real-life social disconnection and the parasocial relationship. Correspondingly, the components most likely to couple with these real-life deficits in the parasocial relationship are sustained attention to the idol's life, tendencies to discuss them, and emotional involvement. Thus, the connection between loneliness and the parasocial relationship between virtual idols and fans exhibits clear local bridging characteristics rather than a simple overall covariation.

Regarding result robustness, the correlation stability coefficient for Strength was 0.75, reaching the highest level within the current testing range, indicating that the centrality results have high stability. The core and bridging nodes presented in Figure 4 are not merely products of random fluctuations but are grounded in a reliable statistical foundation. The key node structure identified in this study demonstrates good replicability, providing a solid basis for further exploring how loneliness is embedded within the parasocial relationship between virtual idols and fans at the micro-component level.

Given that the previous results indicated that gender moderated the statistical associations among physical activity, loneliness, and parasocial relationships with virtual idols, this study further conducted an exploratory network comparison by gender. This study further conducted an exploratory network comparison based on gender. The male sample consisted of 1,472 participants, and the female sample consisted of 1,326 participants. The network comparison results indicated no statistically significant differences in the overall structure of the networks between the two groups. The network invariance test statistic was 0.145, with a *p*-value of 0.118. There were also no significant differences in the overall connection strength, with global strength values of 10.27 for the male group and 9.96 for the female group. The global strength invariance test statistic was 0.312, with a *p*-value of 0.535. These results suggest that, although females exhibit higher sensitivity in the relationships between the aforementioned variables, this difference is primarily reflected in the intensity of the effects at the variable level and has not led to a significant reorganization of the loneliness and parasocial relationship between virtual idols and fans item network structure.

## 4 Discussion

### 4.1 Summary of key findings

Against the backdrop of the continued weakening of real-life social connections and the growing emotional needs of youth directed toward media spaces, the parasocial relationship between virtual idols and fans is no longer merely a media phenomenon, but rather a socio-psychological issue involving the absence of real-life connections, emotional compensation, and psychological adaptation. As virtual idols increasingly become objects of emotional projection for youth, a more pressing question is not whether individuals form parasocial relationships with virtual idols, but whether protective experiences in real life are associated with such compensatory emotional involvement and how these associations are reflected in specific psychological patterns. This study found that higher levels of physical activity were associated with weaker parasocial relationships with virtual idols, and this association was partially statistically mediated through lower levels of loneliness. Furthermore, gender moderates this set of associations, with females demonstrating higher sensitivity in the relevant pathways. However, this difference primarily manifests in the strength of the associations between the variables, rather than in a significant reorganization of the overall item network structure. Item network analysis also suggested that the key links connecting loneliness and parasocial relationships between virtual idols and fans are the absence of companionship, feelings of neglect, and insufficient relational presence in real life. This suggests that understanding parasocial relationships requires attention not only to the virtual idol itself, but also to the individual's real-life connection experiences and subjective feelings.

### 4.2 Associations among physical activity, loneliness, and parasocial relationships with virtual idols

The results suggest that the association between physical activity and parasocial relationships with virtual idols was partly accounted for by loneliness as a statistical pathway. Higher levels of physical activity are associated with lower levels of loneliness, and higher levels of loneliness are associated with stronger parasocial relationships. This finding is consistent with existing research showing that loneliness is positively associated with parasocial relationships between fans and virtual idols (Liu, 2025). Additionally, a systematic review of the relationship between physical activity and loneliness suggests that physical activity interventions can reduce loneliness under certain conditions (Ahn et al., 2024; Park et al., 2025). This result implies that the link between physical activity and weaker parasocial relationships with virtual idols is not due to physical activity merely occupying time or diverting attention from media, but because they alter the way individuals connect with the real world. For youth, the appeal of parasocial relationships with virtual idols lies significantly in their ability to offer relatively stable, low-risk, and sustainable emotional responses. When real-life experiences of companionship, understanding, and belonging are insufficient, such one-sided yet emotionally involved relationships are more likely to serve a compensatory function (Hoffner and Bond, 2022). In contrast, physical activity, particularly those that are participatory, interactive, and context-dependent, tend to re-embed individuals in real-life situations of bodily engagement, social contact, and emotional coordination, thus reducing the tendency to direct emotional needs toward virtual objects.

This finding aligns with the basic tenets of Self-Determination Theory, which emphasizes that an individual's positive adaptation depends not only on behavior participation but also on whether the behavior fulfills needs for relatedness, competence, and autonomy (Ryan and Deci, n.d.). The significance of physical activity lies in their ability to provide real interaction, group belonging, and positive feedback, rather than merely being a physical expenditure (Teixeira et al., 2012). When individuals consistently experience acceptance, being seen, and shared involvement with others in physical activity, the sense of connection to real-life relationships is strengthened, making it less likely for loneliness to persist or escalate (Ahn et al., 2024). Therefore, the negative association between physical activity and loneliness observed in this study is consistent with the theoretical expectation that physical activity may promote real-life connections and relational fulfillment.

The mediating role of loneliness further reveals the psychological mechanism underlying the formation of parasocial relationships with virtual idols. Loneliness is not equivalent to objective solitude; it refers to an individual's subjective experience of insufficient real-life relationships, making it a stronger predictor of why individuals turn to media relationships for emotional compensation than simply social frequency (Hawkley and Cacioppo, 2010; Kardefelt-Winther, 2014). From the perspectives of belonging needs and compensatory media use, when intimacy, companionship, and social integration are lacking in real life, individuals are more likely to rely on media objects to alleviate discomfort caused by relationship deficiencies (Baumeister and Leary, 1995). The reason virtual idols are particularly suited to serve as compensatory objects is precisely that their images are stable, interactions are sustainable, and emotional projection costs are low (Yu et al., 2025). The results of this study suggest that higher levels of physical activity were associated with lower loneliness, while higher levels of loneliness were associated with greater emotional attachment to virtual idols. In other words, physical activity may not be associated with lower interest in virtual idols *per se*, but may be related to a lower psychological need to compensate for real-life relational deficiencies through parasocial relationships.

### 4.3 Moderating role of gender

This study found that gender is not a mere subsidiary factor in this process, but rather a significant influence on the strength of the relationship between physical activity, loneliness, and the parasocial relationship between virtual idols and fans. Specifically, gender significantly moderated the association between physical activity and loneliness, as well as the association between loneliness and parasocial relationships with virtual idols. Simple slope analysis further revealed that these two pathways are more sensitive in females. That is, compared to males, physical activity showed a stronger negative association with loneliness among females, while loneliness showed a stronger positive association with parasocial relationships among females. This suggests that gender differences are not primarily related to the likelihood of forming parasocial relationships, but more to how real-life experiences are transformed into subjective relationship experiences, and how these experiences further influence emotional investment in virtual idols (Cross and Madson, 1997).

This result can be understood from the perspectives of gender socialization and relationship orientation. Existing research generally suggests that different genders exhibit differences in emotional expression, interpersonal cue processing, and relationship-building methods (Kret and De Gelder, 2012). In comparison, females typically place greater importance on relationship quality, emotional responsiveness, and interpersonal connections, making them more sensitive to feelings of support, companionship, and belonging in real-life relationships (Cross and Madson, 1997). Under this premise, the significance of physical activity for females may lie not only in physical participation but also in whether they provide real interaction, feelings of acceptance, and group belonging. When these positive experiences are reinforced in physical activity, females are more likely to experience a reduction in loneliness. In the absence of real-life connections, loneliness may be more strongly linked to parasocial relationships with virtual idols among females for stable emotional responses and psychological comfort, consistent with the findings of Ahn et al. (2024) and Eather et al. (2023).

This finding also suggests that the psychological significance of physical activity may differ between genders. For females, the social interaction, relational support, and sense of belonging inherent in physical activity may offer greater explanatory power than simply the frequency of participation (Laird et al., 2016). In other words, the stronger association between physical activity and lower loneliness among females may not be attributable only to more frequent participation, but may also reflect females' greater sensitivity to the interactive atmosphere, joint participation, and emotional connections within physical activity. Correspondingly, when real-life connections are insufficient, loneliness may be more strongly associated with parasocial relationships with virtual idols. Virtual idols can provide continuous attention, imaginative interactions, and lower-risk emotional investment, making them an easily accessible emotional resource for individuals with a stronger relationship orientation and greater emotional feedback needs.

### 4.4 The value of item network and bridging nodes

To further explore which specific psychological components drive the parasocial relationship between virtual idols and fans, it is essential to move beyond relying on total scores, which often compress the inherent heterogeneity of the internal structure into a single indicator, weakening the ability to identify the specific mechanisms at play (Fried and Nesse, 2015). The value of item network analysis lies in its ability to further break down the relationships between variables into the relationships between components, thereby revealing which experiences are more likely to serve as key points connecting the absence of real-life relationships with emotional investment in virtual idols. The network results of this study indicate that loneliness and the parasocial relationship between virtual idols and fans do not merge into a single structure, but instead form two relatively distinct components that are connected through a few key bridging nodes. Notably, the nodes with the strongest bridging effects are concentrated in the experiences of loneliness, such as “lack of companionship,” “feeling neglected,” and “others are around but not truly with me,” as well as parasocial investment components such as “talking about her” and “caring about her life.” This result suggests that it is not the abstract concept of loneliness as a total score, but rather subjective experiences directly linked to the absence of companionship, relational neglect, and insufficient presence, that drive individuals from real-life relational deficiencies to emotional attachment to virtual idols. These findings advance our understanding of the underlying mechanisms and further support the conclusion regarding the mediating role of loneliness in earlier sections of this study.

This aligns with theories on network structure, belonging needs, and compensatory media use. Existing theories suggest that when individuals struggle to obtain sufficient support, understanding, and connection in real-life relationships, they are more likely to turn to media relationships to meet their emotional needs (Baumeister and Leary, 1995; Borsboom and Cramer, 2013; Kardefelt-Winther, 2014). The network results in this study further show that it is not general negative emotions, but rather specific loneliness experiences with clear relational attributes, that are closely associated with the parasocial relationship. This implies that individuals are more likely to rely on virtual idols not due to simple emotional distress, but because they feel a lack of companionship and response in real life, or even perceive others as being present without truly connecting with them. In such cases, individuals are more likely to compensate for deficiencies in real-life relationships through sustained attention and emotional investment in virtual idols. At the same time, the ability of physical activity to mitigate this process is not merely due to their physical activity attribute. More importantly, physical activity provide real-life interaction, participation, and a sense of belonging, which primarily address subjective experiences directly related to relational deficiencies. Therefore, the findings of this study do not suggest a general relationship between loneliness and parasocial relationships, but rather a more specific psychological pathway: the lack of real-life relational experiences, especially the absence of companionship and acceptance, is more likely to drive individuals to seek emotional compensation through parasocial relationships with virtual idols.

### 4.5 Theoretical significance and practical implications

This study approaches parasocial relationships with virtual idols from the perspective of real-life lifestyles, extending the traditional focus on media attributes and idol characteristics. The results suggest that parasocial relationships with virtual idols are not isolated media phenomena but are closely related to adolescents' real-life engagement, relational experiences, and emotional needs. Physical activity were associated with these parasocial relationships partly through lower loneliness, and the network analysis further indicated that this association was linked to specific experiences such as lack of companionship, feelings of neglect, and emotional involvement. From a practical standpoint, physical activity has already been incorporated into regular school education in China through the Physical Education and Health curriculum, and national policies require schools to fully implement physical education courses. Therefore, interventions should not focus only on limiting media use, but should also improve adolescents' actual participation in physical activity and real-life interactions. Schools can enhance participation by ensuring sufficient physical education time, improving class quality, organizing after-school sports, and increasing cooperative or team-based activities. Families can support adolescents by encouraging regular exercise, participating in activities with them, providing emotional support, and helping them balance screen-based activities with offline engagement. Future research should further examine how school physical education, extracurricular sports, family support, and different types of physical activity are associated with loneliness and parasocial relationships with virtual idols, preferably through longitudinal or intervention designs.

### 4.6 Research limitations and future directions

This study has several limitations. First, the cross-sectional design limits the ability to determine causal directions between the variables. Although this study found that higher levels of physical activity were associated with weaker parasocial relationships with virtual idols through lower loneliness, reverse or reciprocal pathways cannot be ruled out. For example, stronger parasocial relationships with virtual idols may increase adolescents' loneliness by replacing or weakening real-life social interactions, and may also be associated with lower physical activity by occupying time, attention, and emotional investment that could otherwise be directed toward offline activities. Therefore, the directionality and causal nature of these associations require further validation through longitudinal, cross-lagged, or intervention studies. Second, the study predominantly used self-report questionnaires to collect data, which may still be subject to subjective biases. Third, the item network analysis reveals conditional associations rather than causal relationships in the strictest sense; thus, interpretations of the bridging nodes should be approached with caution. Future research can further explore the dynamic relationships between physical activity, loneliness, and parasocial relationships with virtual idols using longitudinal tracking or experimental intervention designs. Additionally, incorporating variables such as social support, peer relationships, and school belonging will provide a more comprehensive understanding of the role of real-life connections in these processes. It is also necessary to focus on the differences between various gender groups and developmental stages to offer more targeted evidence for promoting adolescent mental health and repairing real-life relationships.

## Data Availability

The raw data supporting the conclusions of this article will be made available by the authors, without undue reservation.
